# Evaluation of two eco-friendly neutralizers for a spectrum of tissue fixatives for biomedical applications

**DOI:** 10.4155/fsoa-2018-0035

**Published:** 2018-08-10

**Authors:** Roshini Prakash, Stanley Thomas Carmichael

**Affiliations:** 1 Department of Neurology, David Geffen School of Medicine, University of California, Los Angeles, CA, USA

**Keywords:** aldehyde toxicity, formaldehyde inactivation, glutaraldehyde inactivation, tissue fixatives

## Abstract

Formaldehyde is a widely used aldehyde in biomedical applications, including tissue fixation. It is this same fixative property that can result in toxicity if aldehydes are improperly discarded. A proper neutralization of aldehyde waste products can address this, thereby reducing both health and environmental toxicity concerns. In this study two commercially available products designed to neutralize formaldehyde were evaluated, including neutralization of laboratory derived tissue fixative waste. The primary selection criteria for inclusion in the study were: their ease of use (based on product instructions); the two products assert high levels of formaldehyde neutralization (below 20 ppm) relative to other neutralizing products and their lack of generation of polymeric residues that can clog drains. Both products tested were relatively easy to use and both achieved <10 ppm residual levels of formaldehyde from standard formalin and glutaraldehyde preparations used in research and clinical laboratories.

## Lay abstract

Formaldehyde is widely used as a tissue fixative (preservative) in the biomedical sciences. It unfortunately has an associated toxicity that can manifest as health and environmental hazards, which has led to the creation of restrictions in the disposal of formaldehyde waste. In this study two commercially available products designed to inactivate formaldehyde (and glutaraldehyde wastes) were evaluated for ease of use and ability to reduce detectable formaldehyde levels. Both products tested (FormaGO^®^ and Neutralex^®^) were relatively easy to use and both achieved residual formaldehyde and glutaraldehyde levels (<10 ppm) with formaldehyde and glutaraldehyde preparations at concentrations typically used in laboratories.

Formaldehyde gas and its aqueous solution, also known as formalin, are used in a broad range of commercial applications. The global production of formaldehyde was over 46.7 million tons in 2012 and was expected at that time to surpass 52 million tons by 2017 [1]. Although a majority of the formaldehyde manufactured is used in polymer manufacturing processes [2,3], much of it is used in fields that generate significant amounts of waste formalin. These include paper and textile manufacturing, the fisheries industry and a range of biomedical disciplines. Examples of the latter include clinical sterilization and disinfection processes, embalming procedures at mortuaries and the preservation of biological samples in pathology laboratories, research institutions and museums. Another aldehyde, glutaraldehyde, is also widely used commercially and finds similar applications such as disinfection and sterilization, tissue fixation and water treatment.

With the widespread use of these aldehydes comes notoriety due to their toxic natures and corresponding health and environmental concerns upon disposal. Formaldehyde is classified as a carcinogen (category 1A), germ cell mutagen (category 2), skin sensitizer (category 1), target organ toxin-single exposure (category 1), acute toxin-oral, dermal and inhalation (category 3) and toxic to aquatic life (category 3) [4–7]. Although glutaraldehyde is not classified as a carcinogen, it is still classified as respiratory and skin sensitizer (category 1), corrosive to the skin (category 1B), acute toxic to aquatic life (category 1), chronic toxic to aquatic life (category 2) and acute oral and inhalation toxin (category 3) [8].

As with other compounds classified as hazardous, the properties of these two chemicals raise concerns about their disposal after use [9]. Both the scientific literature and the design of many industrial products (whether actual or described in patents) suggest some compounds can render hazardous formalin or glutaraldehyde solutions into nonhazardous waste that is safe to dispose into the environment without toxic effects [10–14]. The purpose of this study was to evaluate two such commercial products to determine their effectiveness in neutralizing aldehydes (and their waste) used extensively in tissue fixation and sterilization. Two formaldehyde neutralizers which suggests residual formaldehyde levels below 20 ppm and the absence of solid polymeric waste production were chosen: Neutralex^®^ (Scigen Inc., CA, USA) based on its California Department of Toxic Substance Control certification [19] and a more recently introduced product, Tissue–Tek FormaGO^®^ (Sakura Finetek USA Inc., CA, USA) [14,15]. An additional consideration was ease of use featured by both neutralization products.

## Methods

Total 10% formalin solutions (3.7% formaldehyde aqueous solution) from both before and after tissue fixation were used for testing of the neutralizing products. The subject solution to be neutralized consisted of buffered and unbuffered forms of formalin in both used and unused forms. Neutral buffered formalin (10% NBF) was obtained from Medical Chemical Corporation, CA, USA. Used formalin refers to formalin used for for fixation of animal tissues by immersion of the tissues in formalin at 1:10 v/v tissue:formalin ratio for minimum of 12 h. Additionally, 2.5% glutaraldehyde (Wavicide^®^, Medical Chemical Corporation, CA, USA), used to sterilize surgical instruments, was tested before and after use. Each group was tested with n = 6 replicates.

## Neutralization procedure

All the solution groups were neutralized using either Neutralex or Tissue–Tek FormaGO as per each manufacturer's instructions [14–17]. Neutralization was performed inside the fume hood, with regular swirling of the container until the neutralization salts were thoroughly dissolved and the solution appeared clear. After neutralization, solutions were tested for the amount of original product left in solution using semiquantitative test strips.

## Measuring neutralization capacity

Neutralization capabilities were assessed in an unbiased way (i.e., using test materials obtained from a third party, not those provided with the neutralization reagents) through the use of Quantofix^®^ Formaldehyde test strips (Machery-Nagel, PA, USA). Each solution's pH was also tested following neutralization using pH strips (Machery-Nagel). Quantofix Formaldehyde test strips are scaled semiquantitatively on a scale of 0–10–20–40–60–100–200 ppm, with colors ranging from off-white to gradients of purple. Each neutralized sample to be tested was filled in the cuvette to the appropriate level marked by the manufacturer (∼5 ml). Ten drops of the activating reagent were added and mixed with the sample by swirling. After 30 s, the solution was tested with the test strips provided with the test kit by dipping the test strip into the solution for 1 s, shaking off excess reagent, and waiting for exactly 60 s before reading the results. All solutions and their accompanying waste were disposed appropriately as biohazardous formaldehyde waste. To measure glutaraldehyde concentration post neutralization, Quantofix Glutaraldehyde test strips (Machery-Nagel) were used in a similar manner.

## Results

### Neutralization effectively depleted formaldehyde from formalin solutions

Neutralization of formalin in laboratories is an effective way to reduce formaldehyde toxicity. To test the efficiency of the neutralization, we collected small quantities of formalin that had been used for fixation of animal tissues in the course of several investigational procedures. Similar amounts of unused formalin were also set aside for testing. An appropriate amount of neutralizing product (FormaGo or Neutralex) was added to each sample, thoroughly mixed and incubated as recommended in the corresponding product data sheet (15 min for Neutralex and 5 min for FormaGo). After the recommended neutralization time was complete, the sensitivity of the reaction was tested using the semiquantitative Quantofix test strips. As shown in Tables 1 and 2, both of the neutralizing products tested equally depleted the formaldehyde levels to the ‘0’ ppm level (n = 6 per group) in all trials, and the corresponding pH values ranged from six to eight. Neutralization of 2.5% glutaraldehyde was equally effective with either FormaGo or Neutralex, with both yielding 0 ppm results when tested using glutaraldehyde test strips.

**Table 1.** **Formalin and glutaraldehyde neutralization results for Neutralex^®†,‡^.**

	**10% Formalin**			**2.5% Glutaraldehyde**		

**Parameters**	**Buffered^§^ unused**	**Buffered^§^ used^¶^**	**Unbuffered unused**	**Unbuffered used^¶^**	**Used^#^**	**Unused**
Concentration of formaldehyde after neutralization, ppm	0	0	0	0	0	0

Standard deviation for ppm	0	0	0	0	0	0

pH after neutralization	6.67	7.17	6.83	7.33	7.5	6.67

Standard deviation for pH	0.52	0.41	0.41	0.52	0.55	1.03

^†^Neutralex concentration used was 19.74% w/v of 10% formalin or 2.5% glutaraldehyde solution; based on the manufacturer's recommendation of one bag of 750 gm per 1 gal. of the waste reagent.

^‡^Mixture contact (incubation) time was 15 min as recommended by product literature.

^§^Buffered formalin was obtained as 10% Neutral buffered formalin and it contained phosphate buffer; which is a mixture of sodium phosphate monobasic and sodium phosphate dibasic.

^¶^Used formalin solution was generated by utilizing it for fixation of animal tissues at 1:10 ratio of tissue: formalin by volume for minimum of 12 h.

^#^Used glutaraldehyde solution was generated by utilizing it to sterilize surgical instruments in the laboratory.

**Table 2.** **Formalin and glutaraldehyde neutralization results for Tissue–Tek FormaGO^®†,‡^.**

**Parameters**	**10% Formalin**			**2.5% Glutaraldehyde**		

	**Buffered^§^ unused**	**Buffered^§^ used^¶^**	**Unbuffered unused**	**Unbuffered used^¶^**	**Used^#^**	**Unused**
Concentration of formaldehyde after neutralization, ppm	0	0	0	0	0	0

Standard deviation for ppm	0	0	0	0	0	0

pH after neutralization	7	7	7	6.67	6.5	5.67

Standard deviation for pH	0	0	0	0.41	0.54	0.51

^†^Neutralex concentration used was 19.74% w/v of 10% formalin or 2.5% glutaraldehyde solution; based on the manufacturer's recommendation of 1 bag of 750 gm per 1gal. of the waste reagent.

^‡^Mixture contact (incubation) time was 15 min as recommended by product literature.

^§^Buffered formalin was obtained as 10% neutral buffered formalin and it contained phosphate buffer; which is a mixture of sodium phosphate monobasic and sodium phosphate dibasic.

^¶^Used formalin solution was generated by utilizing it for fixation of animal tissues at 1:10 ratio of tissue: formalin by volume for minimum of 12 h.

^#^Used glutaraldehyde solution was generated by utilizing it to sterilize surgical instruments in the laboratory.

## Discussion

Both neutralizers evaluated in this study, Neutralex and Tissue-Tek FormaGO showed equivalent and comparable results. To further clarify the significance of those results, it is worth noting that neutralization of 10% formalin below 20 ppm is understood to be satisfactory based on the results evaluated by the California Department of Toxic Substance Control in the course of their decision to certify Neutralex as an approved waste treatment technology for formalin. It is difficult to discern precisely what constitutes an ‘acceptable’ concentration of formaldehyde in effluents for disposal, but other commercial products used for the neutralization of formalin claim residual formaldehyde concentrations approaching 100 ppm. Furthermore, most local governing agencies appear to accept neutralized formaldehyde concentrations below 500 ppm for drain disposal [18]. As a technical observation, it should be noted that, although the test strips showed the results equivalent to ‘0 ppm’ of formaldehyde following neutralization, because the measurement scale exists at increments of 0, 10, 20, 40, 60, 100 and 200 ppm, a value of 0 read from the strip should be interpreted as <10 ppm. Nonetheless, achieving values of <10 ppm in all test conditions shows that both products achieved neutralization to levels below all of the potential standards described above.

The individual performing the study reported the additional observation that the FormaGO product was easier to use due to its ease of handling, more rapid dissolution and its quicker neutralization of the 10% formalin relative to Neutralex.

From the safety datasheet of FormaGO it is noted that the product is a mixture of trisodium phosphate and sodium metabisulfite [20]. The former is commonly used as a buffer and the latter is a popular food preservative. With regard to the products of the inactivation, the reaction of formalin and sodium metabisulfite is predicted to result in a sodium formaldehyde bisulfite adduct [21,22] via a mechanism in which sodium metabisulfite first reacts with water to form sodium bisulfite, which in turn reacts with formaldehyde to form the sodium formaldehyde bisulfite adduct [23]. This adduct (CAS:870–72–4) is a stable compound that, according to a representative safety data sheet [24], is not classified as a hazardous substance under OSHA Haz Com: CFR 1910.1200.

Predicted reaction of formaldehyde with sodium metabisulfite:
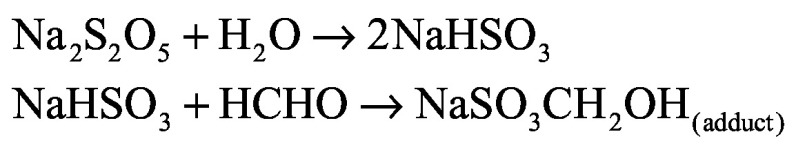



A similar mechanism is predicted for the reaction of sodium bisulfite with glutaraldehyde, and in this regard it is noteworthy that the reaction has been shown to abolish the antimicrobial properties of glutaraldehyde [25]. The anticipated addition product from glutaraldehyde and sodium bisulfite (CAS:7420–89–5) also is classified as nonhazardous product per the safety data sheet [26].

Predicted reaction mechanism of glutaraldehyde with sodium metabisulfite:
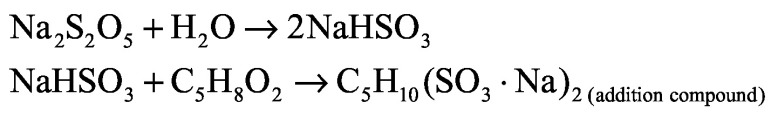



Information regarding the ingredients contained in Neutralex is not available from the manufacturer, and therefore it is not possible to speculate on potential chemical reaction products at this time. Of interest would be studies designed to further characterize the reaction products and document their properties, toxicological and otherwise.

The Neutralex product inset suggests neutralization of 2.5% glutaraldehyde solutions, and our hands it did effectively neutralize the glutaraldehyde solution as claimed. In this study, Tissue–Tek FormaGo neutralizer also effectively neutralized 2.5% glutaraldehyde solutions and to the same level as did the Neutralex product. A similar technical observation regarding the measurement system must be made for the neutralization of glutaraldehyde: just as with the formaldehyde test strips, the glutaraldehyde test strips provide an incremental measurement scale at levels of 0, 0.5, 1, 1.5, 2 and 2.5% glutaraldehyde. This means that readings of 0% glutaraldehyde should be interpreted as <0.5%. This does not, however, obscure the conclusion that equivalent levels of neutralization of glutaraldehyde by both Neutralex and Tissue–Tek FormaGO was achieved.

## Conclusion & future perspective

As previously noted, Neutralex is certified by the California Department of Toxic Substance Control of the California EPA for the effective neutralization of used 10% formalin. The current study has demonstrated that FormaGO neutralized solutions of formalin and glutaraldehyde with efficiencies equivalent to those of Neutralex. This leads us to conclude that both Neutralex and FormaGO neutralizers are suitable for the effective neutralization of 10% formalin and 2.5% glutaraldehyde aqueous solutions as used in both research and clinical laboratories. Depending on specific situations and needs, it may be beneficial in future studies for individual researchers to explore the performance of these products in the context of unique toxicity requirements (e.g., using specific toxicity assays) and/or other methods for the detection of residual aldehydes.

**Summary points**

- Formaldehyde, and to a lesser extent glutaraldehyde, are tissue fixatives broadly used in the biomedical sciences.
- The toxicity associated with both of these aldehydes renders them potential health and environmental hazards, with corresponding restrictions imposed on their use and disposal.
- Two commercially available products (FormaGO^®^, Sakura Finetek USA, Inc., and Neutralex^®^, Scigen, Inc.), designed to inactivate formaldehyde and glutaraldehyde wastes, were evaluated.
- Both FormaGO and Neutralex were found suitable for the effective neutralization of 10% formalin and 2.5% glutaraldehyde aqueous solutions as used in research laboratories.
- Both FormaGO and Neutralex were found to be relatively easy to use.
- The FormaGO and Neutralex products effectively neutralize formaldehyde and glutaraldehyde solutions with residual concentrations less than 10 ppm.
